# Supplementary material for: Efficient biallelic knock-in in mouse embryonic stem cells by in vivo-linearization of donor and transient inhibition of DNA polymerase θ/DNA-PK
Source: Sci Rep. 2021 Sep 13;11:18132. doi: 10.1038/s41598-021-97579-8 (PMC8438075; doi:10.1038/s41598-021-97579-8)
Supplement: Supplementary file 1 — Supplementary Information 1. [file 41598_2021_97579_MOESM1_ESM.pdf]

Efficient biallelic knock-in in mouse embryonic stem cells by *in vivo*-linearization of donor and transient inhibition of DNA Polymerase θ/DNA-PK

Daisuke Arai[1]*, Yoichi Nakao[1,2]

1. School of Advanced Science and Engineering, Waseda University, 3–4–1 Okubo, Shinjuku-ku, Tokyo 169-8555, Japan

2. Research Institute for Science and Engineering, Waseda University, 3–4–1 Okubo, Shinjuku-ku, Tokyo 169-8555, Japan

Corresponding author: Daisuke Arai

Address: 3–4–1 Okubo, Shinjuku-ku, Tokyo 169-8555, Japan

Phone: +81-3-5286-3136

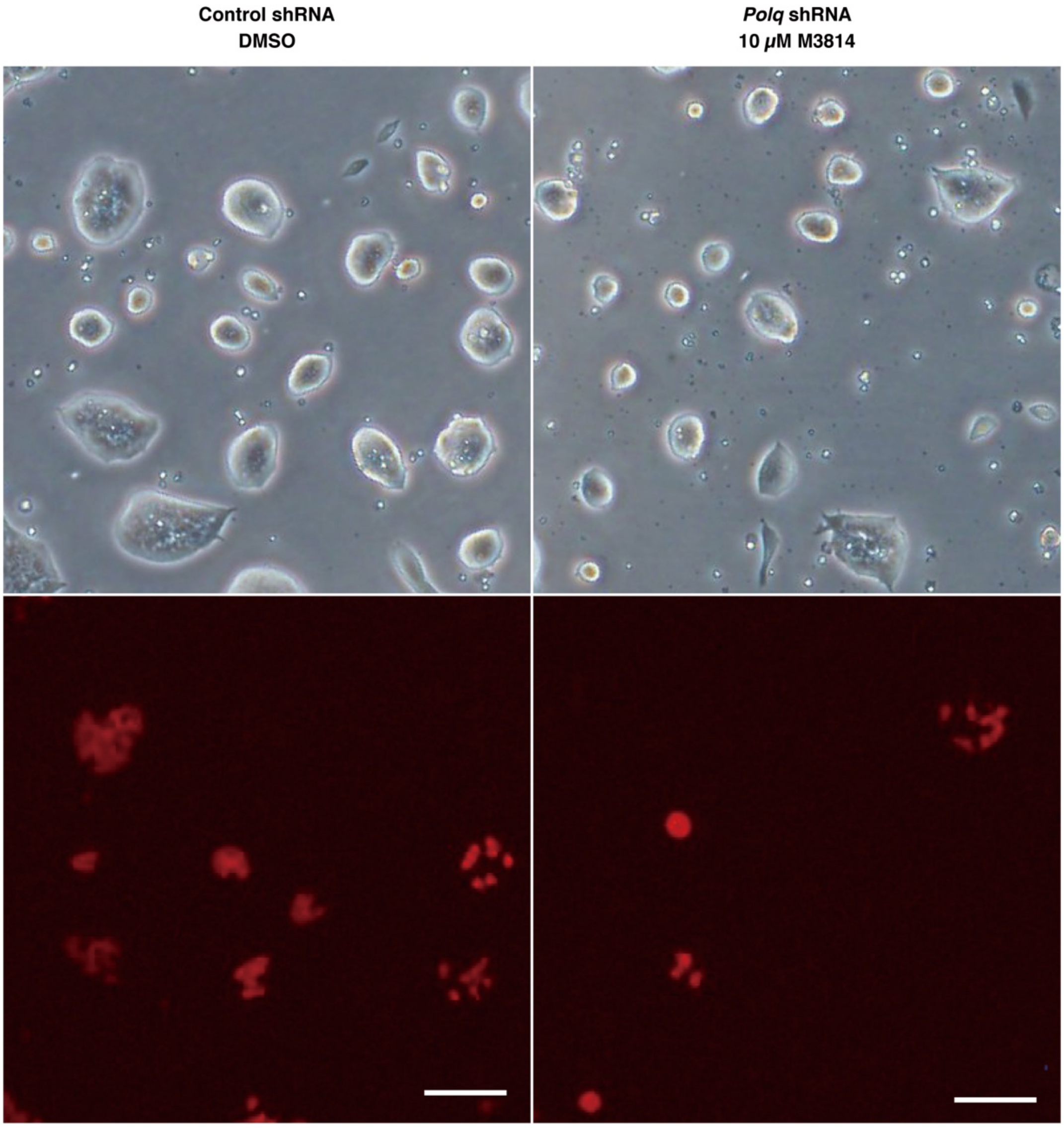

Supplementary figure 1.

Images of control and *Polq* shRNA + 10 μM M3814 cells just before flow cytometry analysis related to Fig. 2d. Scale bar, 100 μm.

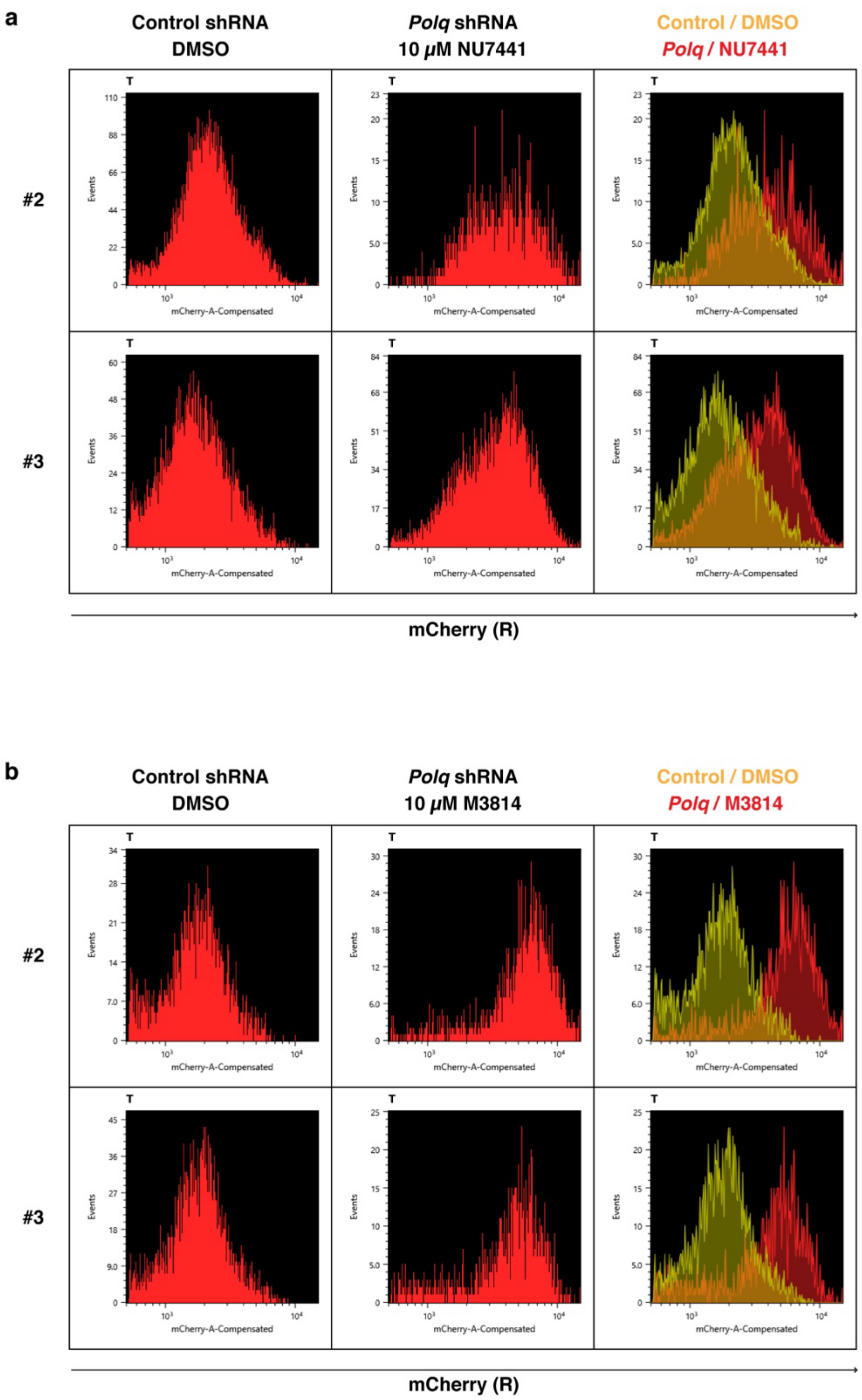

Supplementary figure 2.

Fluorescence intensities of mCherry in R+G– populations from *Polq* shRNA + 10 μM NU7441 (a) and *Polq* shRNA + 10 μM M3814 (b) cells (results not shown in Fig. 3a).

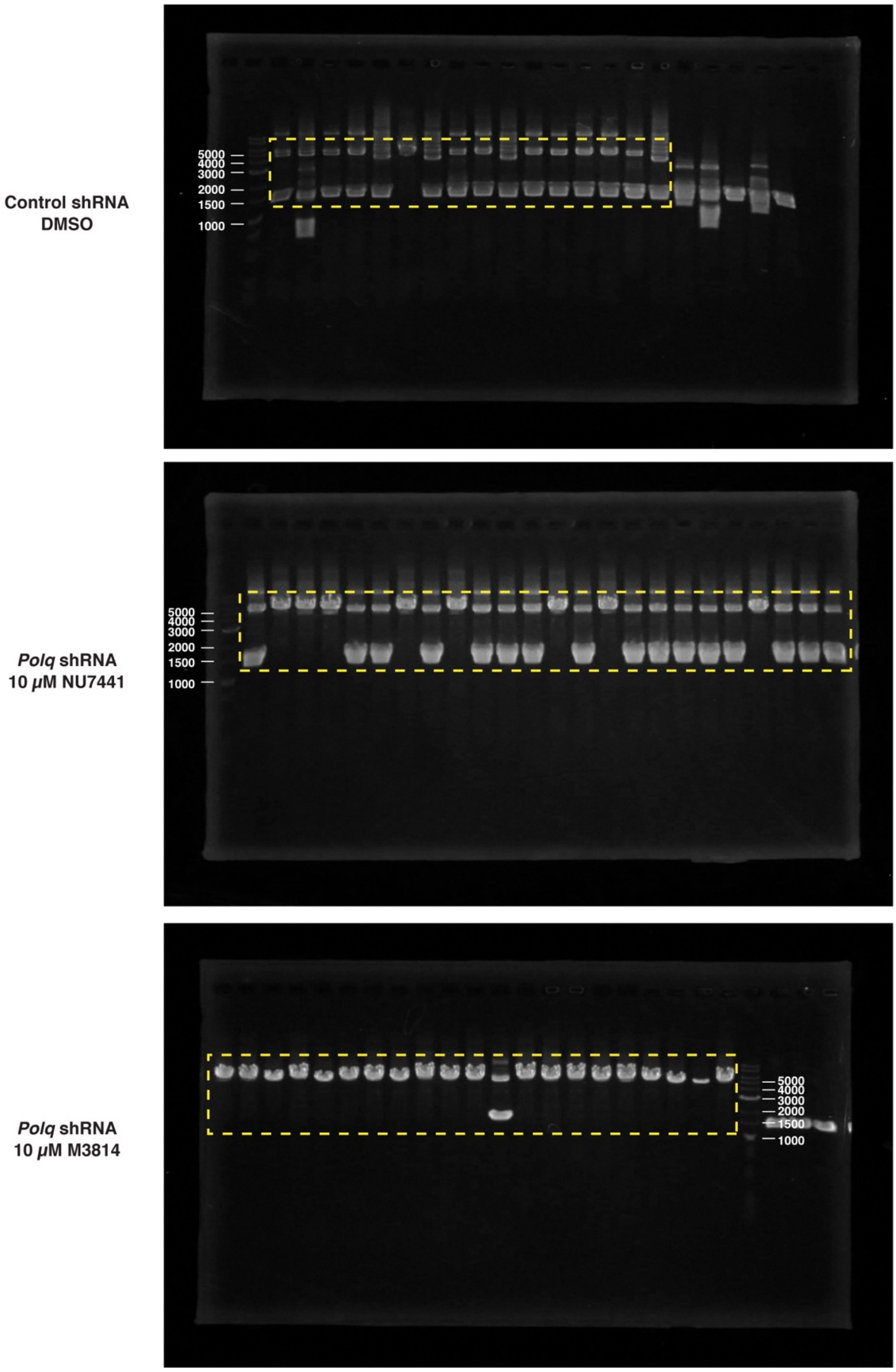

Supplementary figure 3.

Uncropped images with size marker indications related to Fig. 3b.

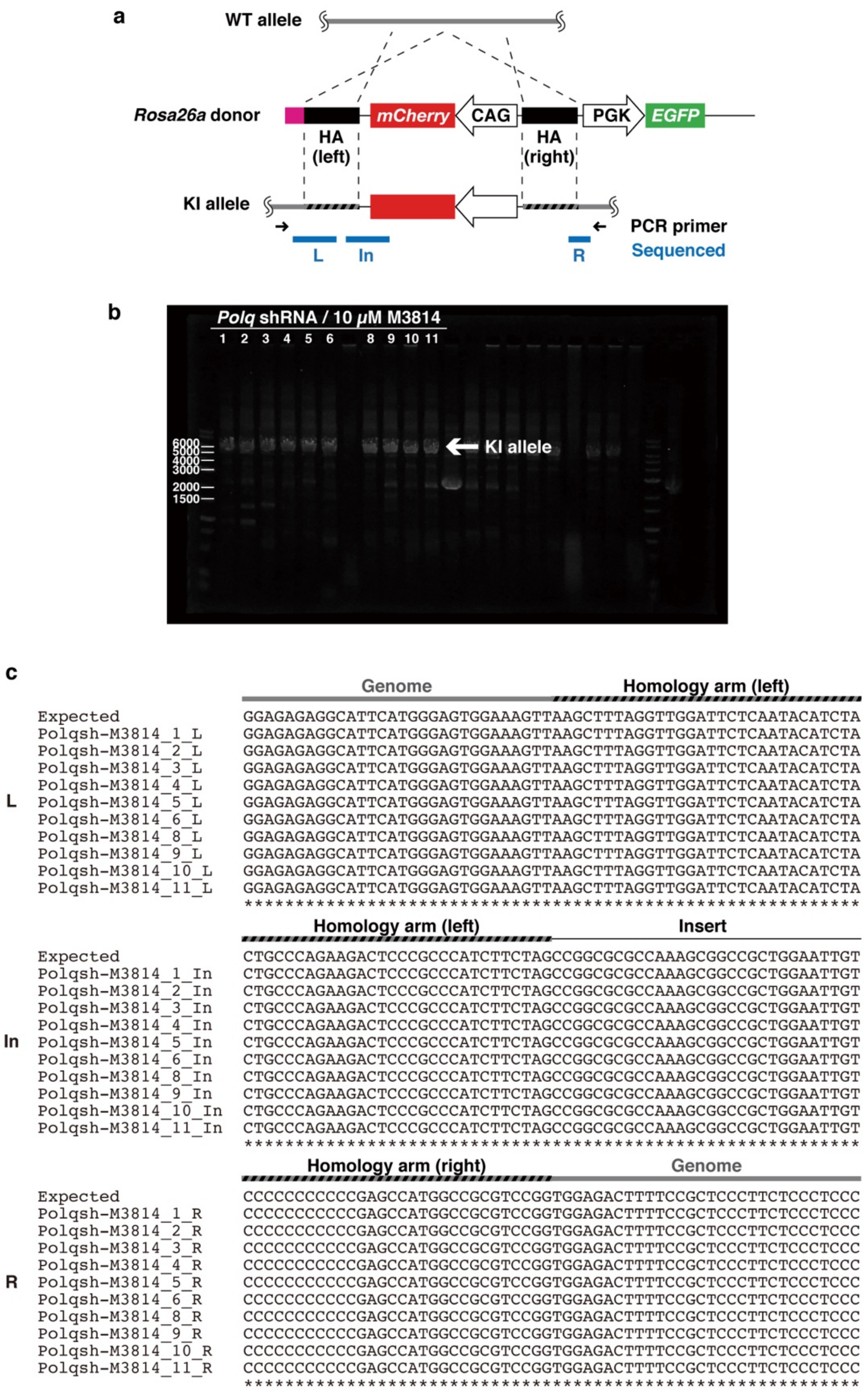

Supplementary Figure 4.

Sequence analysis of *Rosa26a* knock-in alleles from *Polq* shRNA + 10 μM M3814 clones. (a) The position of primers and sequenced regions are shown. (b) PCR products were separated by agarose gel electrophoresis. The uncropped image of the gel is presented. The products of the knock-in

alleles (KI) were purified and analyzed by Sanger sequencing. (c) Sequences are shown for each junction. Clone numbers correspond to those shown in Fig. 3b.

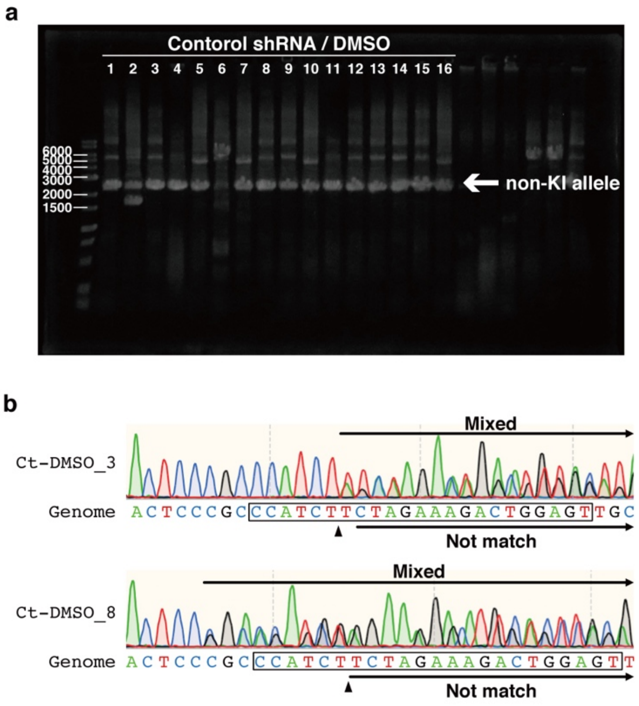

Supplementary Figure 5.

Sequence analysis of *Rosa26a* non-knock-in alleles from control shRNA + DMSO clones related to Fig. 3d. (a) PCR products were separated by agarose gel electrophoresis. The uncropped image of the gel is presented. The products of non-knock-in alleles (non-KI) were purified and analyzed by Sanger sequencing. (b) Sanger sequence traces of clones 3 and 8. The CRISPR target and predicted cut sites are shown by the box and arrowhead, respectively. The PCR products were a mixture of two distinct sequences, suggesting two possibilities. One possibility was that there were two distinct clones present. The other possibility was that the *Rosa26a* locus was duplicated in these clones. In both clones, however, neither sequence matched the wild-type sequence, indicating that there was no wild-type allele present. Clone numbers correspond to those shown in Fig. 3b.

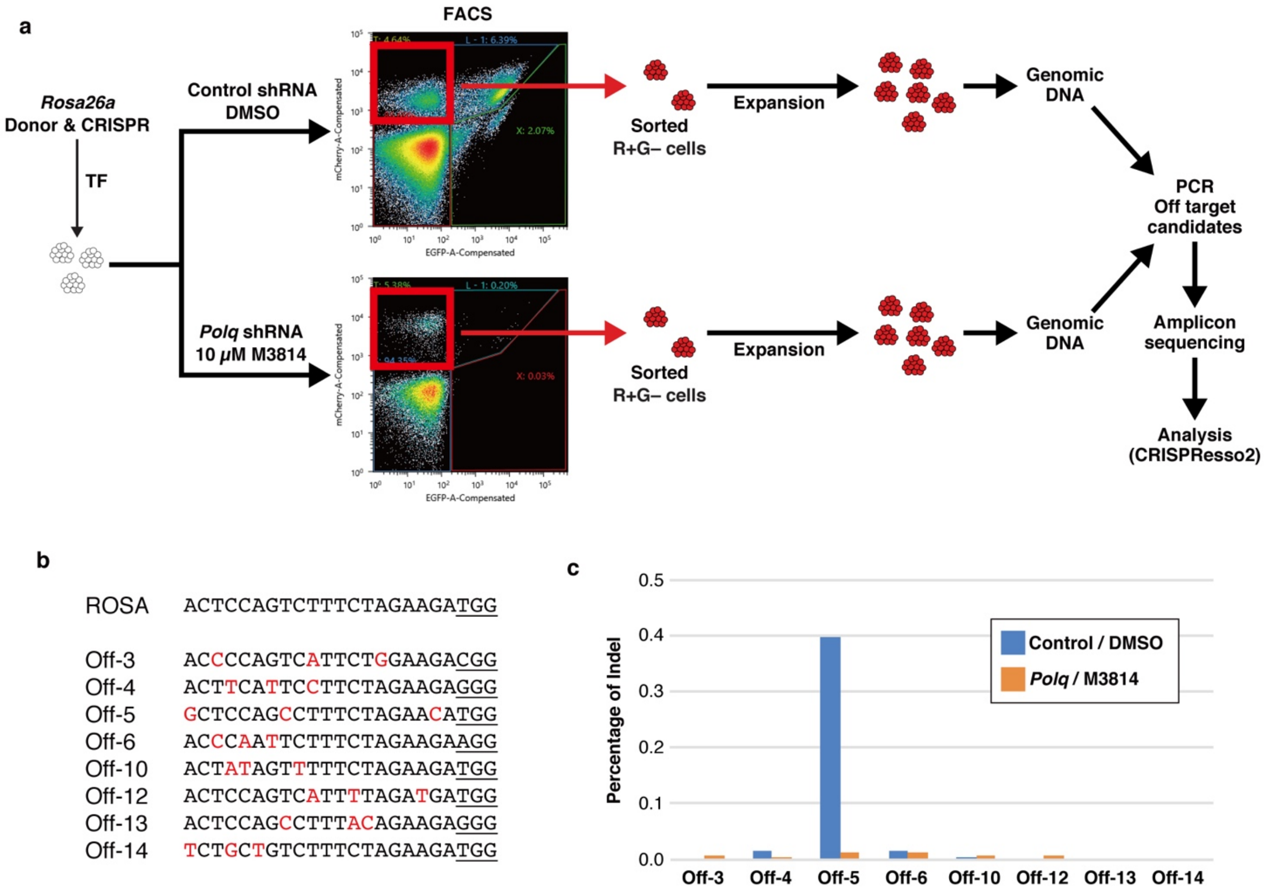

Supplementary Figure 6.

Off-target analysis. (a) Experimental scheme. Actual experimental results of fluorescence-activated cell sorting (FACS) are shown. (b) Sequences of the off-target candidates were examined. (c) Percentage of indel mutations over total reads.

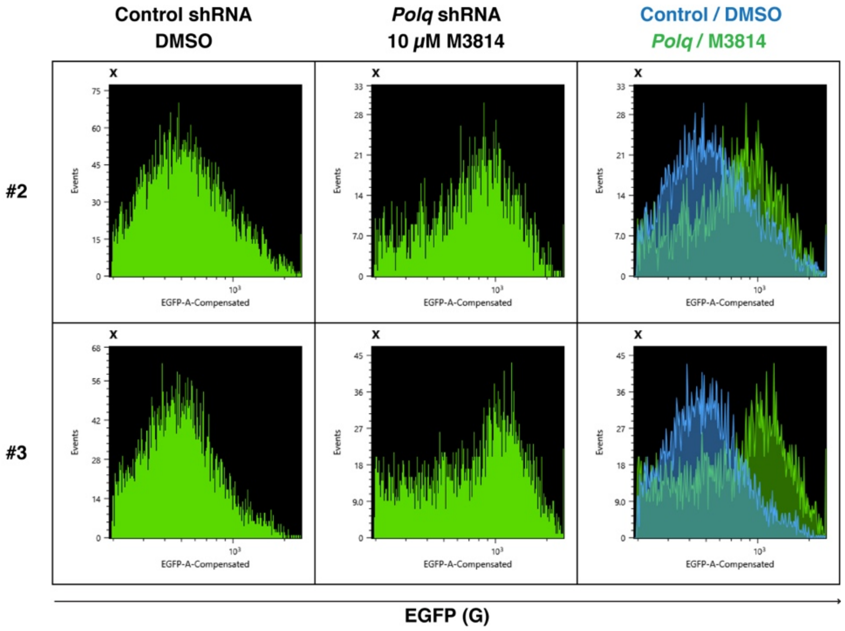

Supplementary figure 7.

Fluorescence intensities of EGFP in R–G+ populations from *Polq* shRNA + 10 μM M3814 cells (results not shown in Fig. 4f).

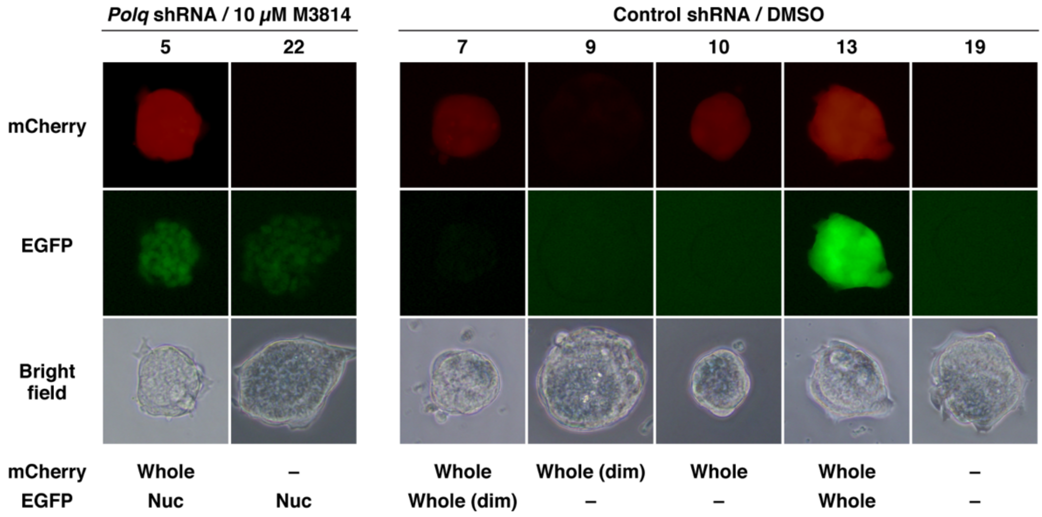

Supplementary figure 8.

Images of isolated clones observed by fluorescence microscopy related to Fig. 5.

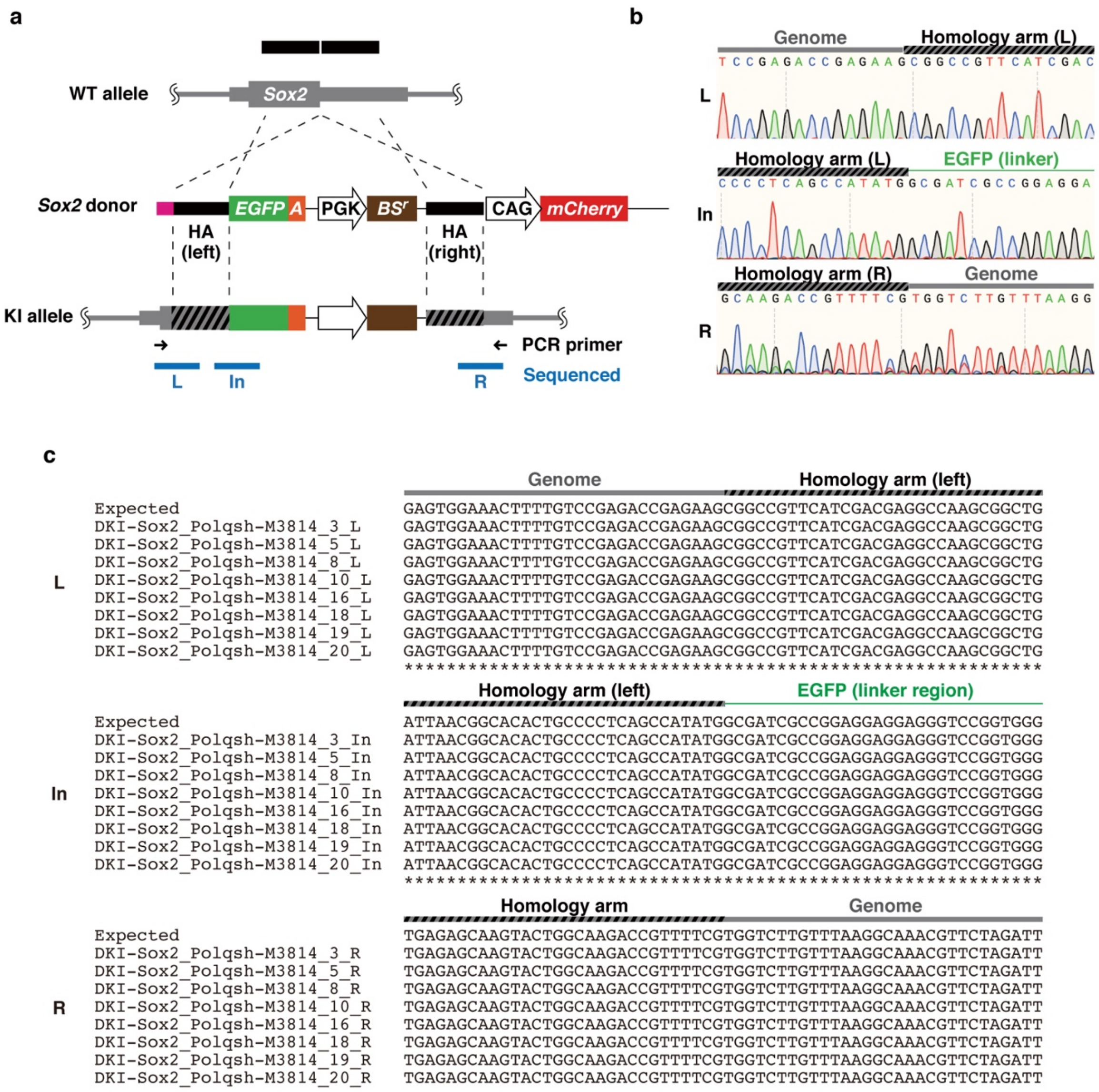

Supplementary Figure 9.

Sequence analysis of *Sox2* knock-in alleles from *Rosa26a-mCherry* and *Sox2-EGFP-mAID* double knock-in clones related to Fig. 5. (a) Position of primers and sequenced regions. (b) Sanger sequence traces of the left junction (L), the junction between the left arm and the foreign sequence (In), and the right junction (R). Representative sequencing data of *Polq* shRNA + 10 µM M3814 clone 3 are shown. (c) Sequences of other *Polq* shRNA + 10 µM M3814 clones.

a

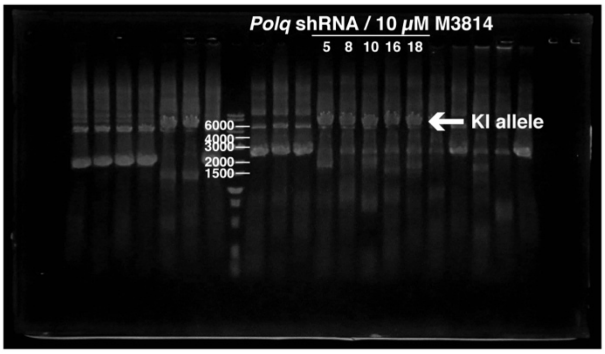

b

```
                                  Genome                     Homology arm
Expected                          GGAGAGAGGCATTCATGGGAGTGGAAAGTTAAGCTTTAGGTTGGATTCTCAATACATCTA
DKI-ROSA_Polqsh-M3814_5_F         GGAGAGAGGCATTCATGGGAGTGGAAAGTTAAGCTTTAGGTTGGATTCTCAATACATCTA
DKI-ROSA_Polqsh-M3814_8_F         GGAGAGAGGCATTCATGGGAGTGGAAAGTTAAGCTTTAGGTTGGATTCTCAATACATCTA
DKI-ROSA_Polqsh-M3814_10_F        GGAGAGAGGCATTCATGGGAGTGGAAAGTTAAGCTTTAGGTTGGATTCTCAATACATCTA
DKI-ROSA_Polqsh-M3814_16_F        GGAGAGAGGCATTCATGGGAGTGGAAAGTTAAGCTTTAGGTTGGATTCTCAATACATCTA
DKI-ROSA_Polqsh-M3814_18_F        GGAGAGAGGCATTCATGGGAGTGGAAAGTTAAGCTTTAGGTTGGATTCTCAATACATCTA
                                  ************************************************************
                                       Homology arm                      Insert
Expected                          CTGCCCAGAAGACTCCCGCCCATCTTCTAGCCGGCGCGCCAAAGCGGCCGCTGGAATTGT
DKI-ROSA_Polqsh-M3814_5_In        CTGCCCAGAAGACTCCCGCCCATCTTCTAGCCGGCGCGCCAAAGCGGCCGCTGGAATTGT
DKI-ROSA_Polqsh-M3814_8_In        CTGCCCAGAAGACTCCCGCCCATCTTCTAGCCGGCGCGCCAAAGCGGCCGCTGGAATTGT
DKI-ROSA_Polqsh-M3814_10_In       CTGCCCAGAAGACTCCCGCCCATCTTCTAGCCGGCGCGCCAAAGCGGCCGCTGGAATTGT
DKI-ROSA_Polqsh-M3814_16_In       CTGCCCAGAAGACTCCCGCCCATCTTCTAGCCGGCGCGCCAAAGCGGCCGCTGGAATTGT
DKI-ROSA_Polqsh-M3814_18_In       CTGCCCAGAAGACTCCCGCCCATCTTCTAGCCGGCGCGCCAAAGCGGCCGCTGGAATTGT
                                  ************************************************************
                                       Homology arm                      Genome
Expected                          CCCCCCCCCCCGAGCCATGGCCGCGTCCGGTGGAGACTTTTCCGCTCCCTTCTCCCTCCC
DKI-ROSA_Polqsh-M3814_5_R         CCCCCCCCCCCGAGCCATGGCCGCGTCCGGTGGAGACTTTTCCGCTCCCTTCTCCCTCCC
DKI-ROSA_Polqsh-M3814_8_R         CCCCCCCCCCCGAGCCATGGCCGCGTCCGGTGGAGACTTTTCCGCTCCCTTCTCCCTCCC
DKI-ROSA_Polqsh-M3814_10_R        CCCCCCCCCCCGAGCCATGGCCGCGTCCGGTGGAGACTTTTCCGCTCCCTTCTCCCTCCC
DKI-ROSA_Polqsh-M3814_16_R        CCCCCCCCCCCGAGCCATGGCCGCGTCCGGTGGAGACTTTTCCGCTCCCTTCTCCCTCCC
DKI-ROSA_Polqsh-M3814_18_R        CCCCCCCCCCCGAGCCATGGCCGCGTCCGGTGGAGACTTTTCCGCTCCCTTCTCCCTCCC
                                  ************************************************************
```

Supplementary Figure 10.

Sequence analysis of *Rosa26a* knock-in alleles from *Rosa26a-mCherry* and *Sox2-EGFP-mAID* double knock-in clones related to Fig. 5. (a) PCR products were separated by agarose gel electrophoresis. The products of non-knock-in alleles (non-KI) were purified and analyzed by Sanger sequencing. (b) Sequences of the homology arms and insert junctions.

Rosa26a

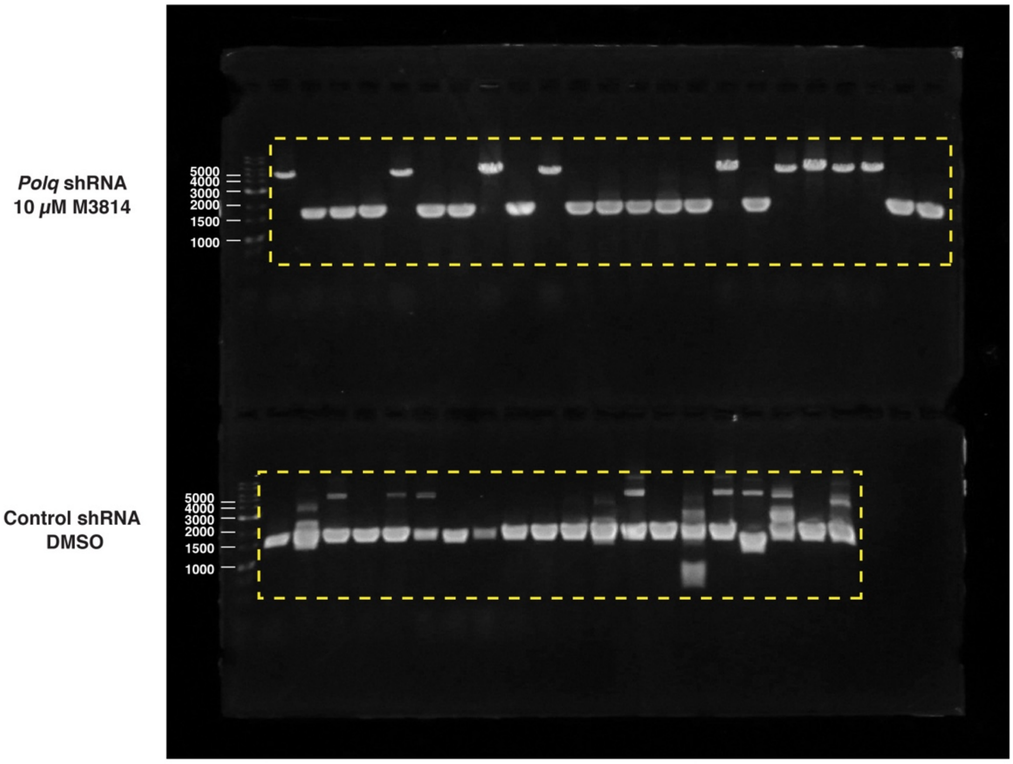

Sox2

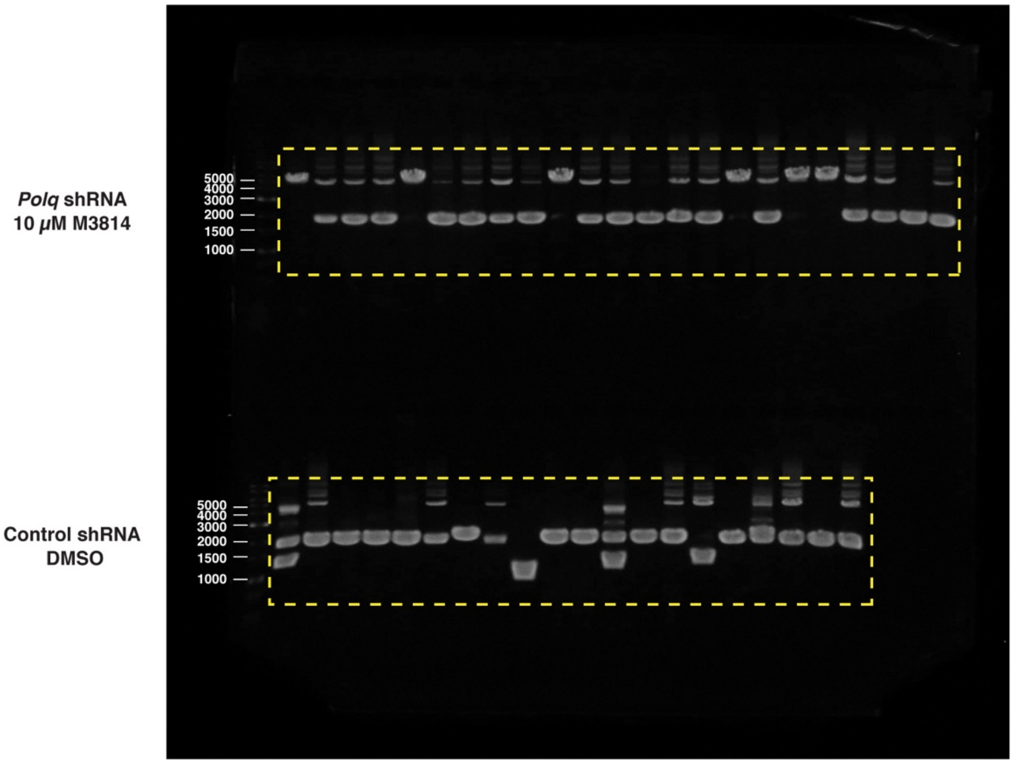

Supplementary figure 11.

Uncropped images with size marker indications related to Fig. 5b and c.

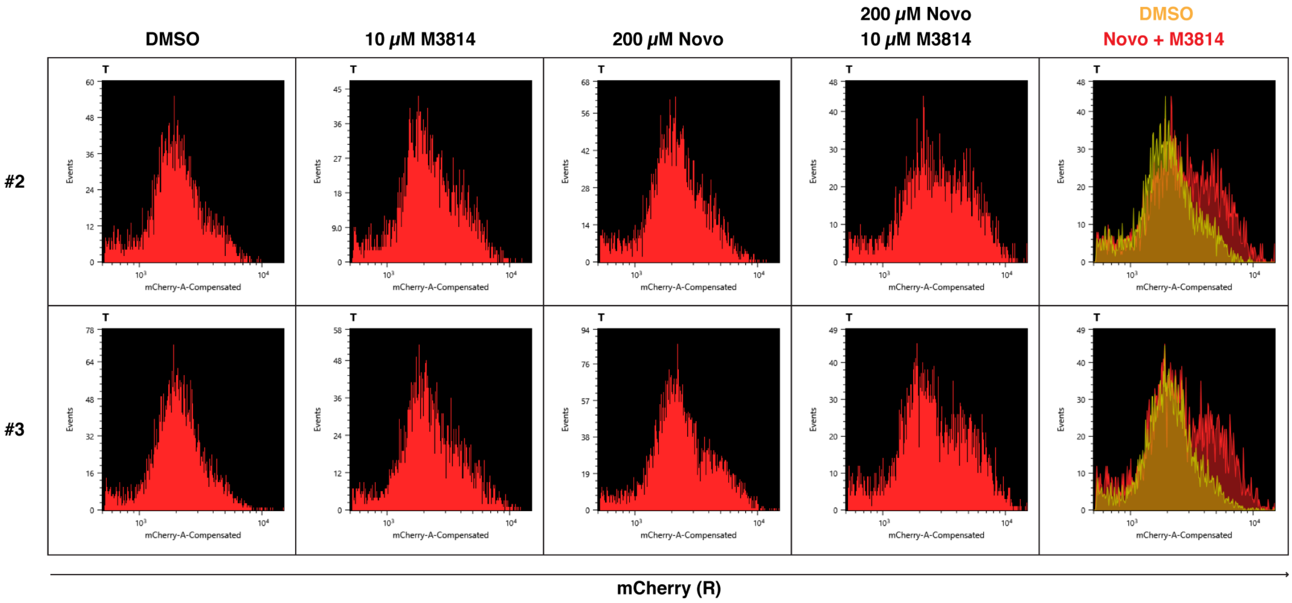

Supplementary figure 12.

Fluorescence intensities of mCherry in R+G– populations from the experiments shown in Fig. 6 (results not shown in Fig. 6c).

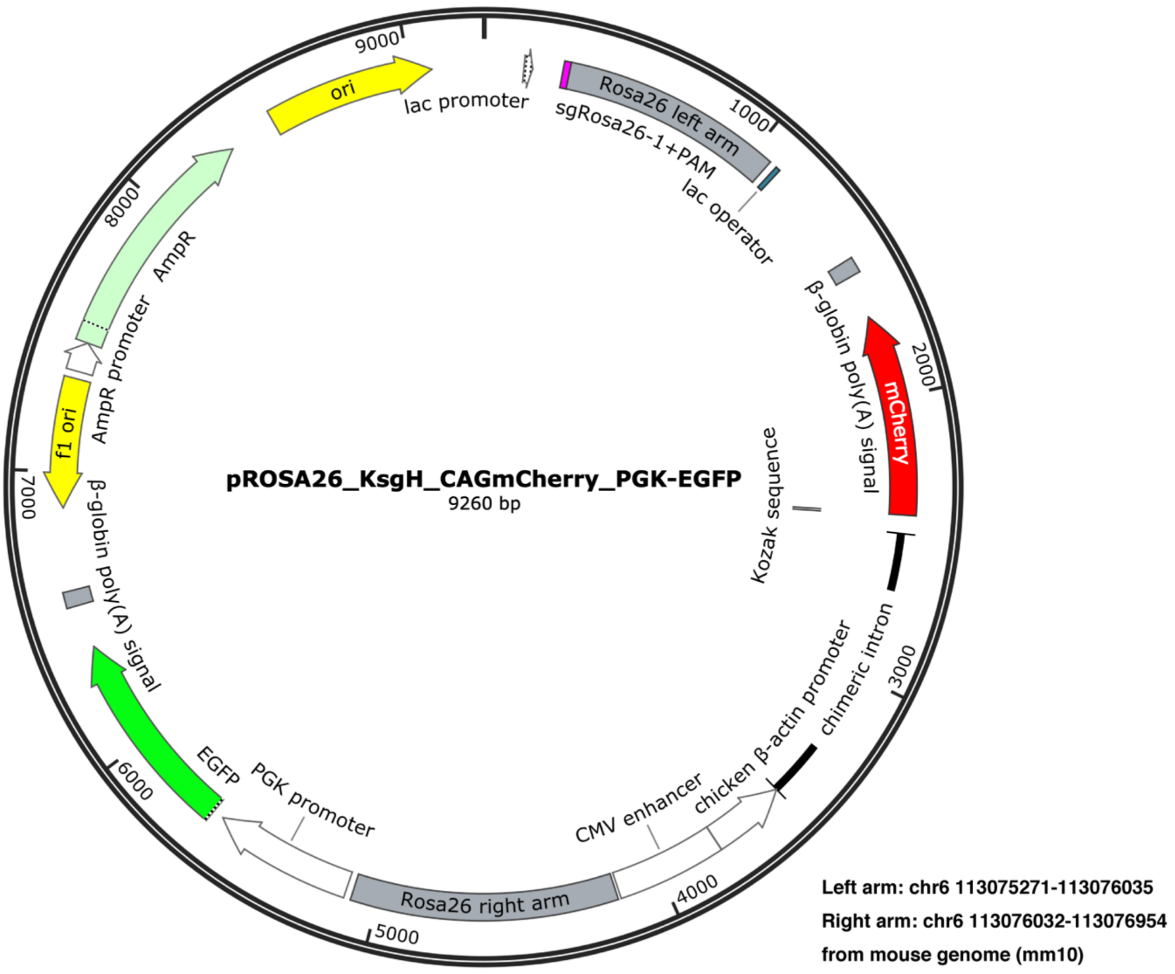

Supplementary figure 13.

Map of the donor plasmid for *Rosa26a-mCherry*.

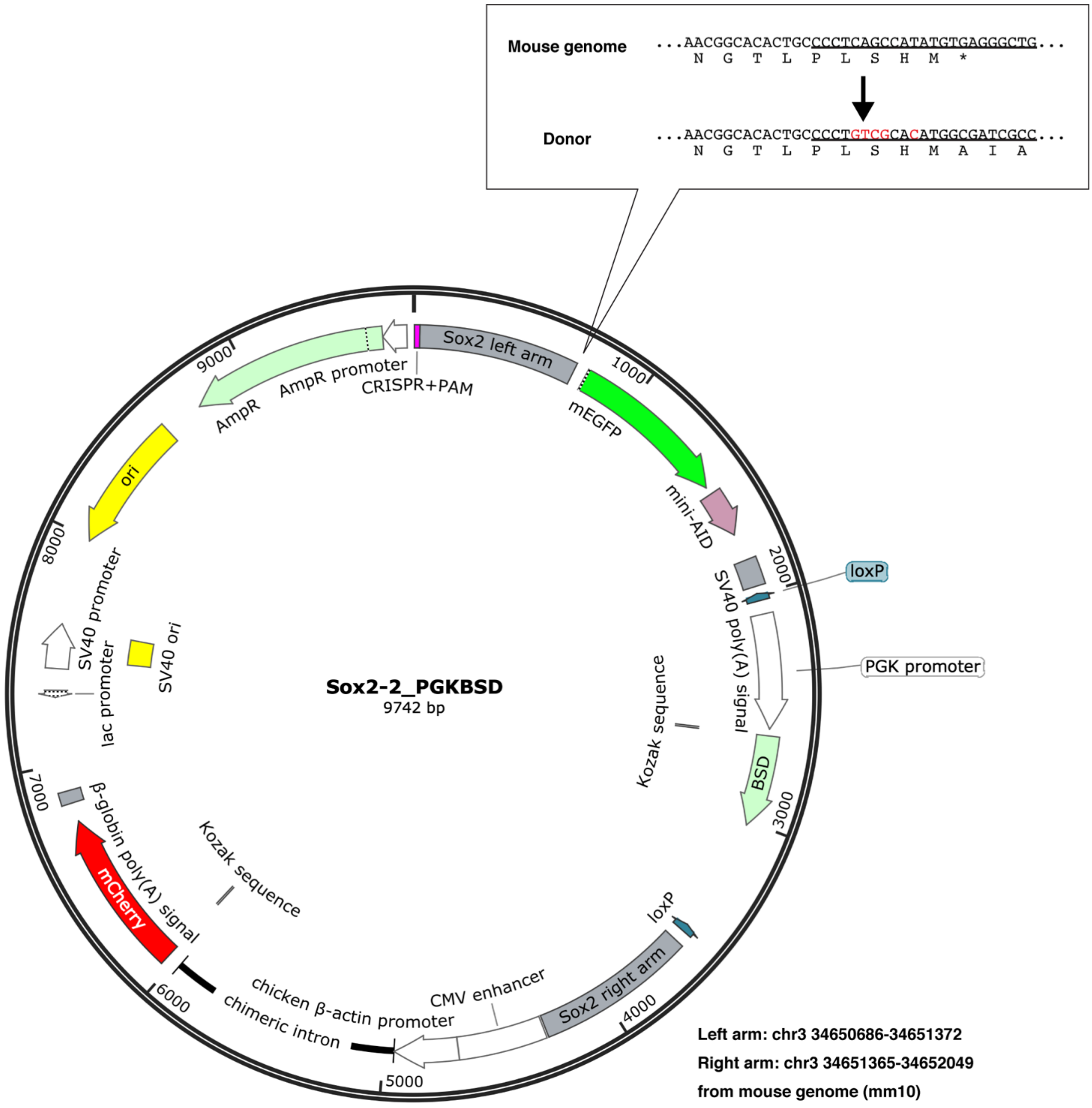

Supplementary figure 14.

Map of the donor plasmid for *Sox2-EGFP-mAID*. The sequence around the *Sox2* stop codon is shown. The target sequence for CRISPR/Cas9 in the mouse genome is underlined.
